# “It's hard to speak up”: Lessons learned from engaging U.S. white college men in conversations on racism

**DOI:** 10.3205/zma001309

**Published:** 2020-03-16

**Authors:** Jörg Vianden, Christian Gruber

**Affiliations:** 1 UW-La Crosse, Department of Student Affairs Administration in Higher Education, La Crosse, USA; 2 Munich, Germany

**Keywords:** racism, masculinity, bystander, joking, dissent, white

## Abstract

**Objective: **This position paper draws on findings from the Straight White College Men Project, a qualitative study with heterosexual white college male participants across college campuses in the United States. The purpose of the larger project was to explore and understand how participants perceived institutional and community diversity issues; how they conceptualized their own privileges; and how they articulated their own responsibility to engage in social change. Thus, this paper delivers “lessons learned” from engaging white men in conversations on racism and to provide recommendations for medical educators in the U.S. and in Europe.

**Methods:** Following purposeful sampling methods using expert nominators, data were collected in phenomenological focus groups at 10 U.S. 4-year universities. Focus groups included 3 to 8 participants and were 60 to 90 minutes in length. Analysis included open and axial coding and two themes emerged from a single question focusing on students’ potential dissent of racist family or peer comments.

**Results: **Participants struggled articulating how and in what contexts they would challenge family members or friends on inappropriate language or behavior. Reasons for their reticence included struggling to confront parents, not wanting to ruin male friendships, or jeopardizing being ousted by the male peer group.

**Conclusion: **Educators must find ways to help male university students explore their privileges and proclivity to engage in oppressive behaviors. Men also need to understand how their inactions perpetuate systemic oppression and create environments in which minoritized individuals cannot thrive. Recommendations for medical school educators are provided.

## Zusammenfassung

**Ziel: **Dieses Positionspapier stützt sich auf Ergebnisse des Straight White College Men Project, einer qualitativen Studie, deren Teilnehmer heterosexuelle, weiße College-Männer an verschiedenen Hochschulen in den USA waren. Der Zweck des Gesamtprojekts bestand darin, zu untersuchen und zu verstehen, wie die Teilnehmer institutionelle und gesellschaftliche Diversitätsprobleme wahrnahmen; welche Vorstellung sie von ihren eigenen Privilegien haben; und wie sie ihre eigene Verantwortung zum sozialen Wandel ausgedrückt haben. So liefert dieses Papier Erkenntnisse aus Gesprächen über Rassismus mit weißen Männer und gibt Empfehlungen für die Ausbildung von Medizinern in den USA und in Europa.

**Methoden:** Nach gezielten Stichprobenverfahren unter Verwendung von Expertennominatoren wurden Daten in phänomenologischen Fokusgruppen an 10 US-amerikanischen 4-jährigen Universitäten gesammelt. Die Fokusgruppen umfassten 3 bis 8 Teilnehmer und dauerten 60 bis 90 Minuten. Die Analyse umfasste offene und axiale Kodierung, und zwei Themen gingen aus einer einzigen Frage hervor, die sich auf den potenziellen Widerspruch der Studenten gegenüber rassistischen Familien- oder Peer-Kommentaren konzentrierte.

**Ergebnisse: **Die Teilnehmer hatten Schwierigkeiten zu artikulieren, wie und in welchen Kontexten sie Familienmitglieder oder Freunde mit deren unangemessener Sprache oder unangemessenem Verhalten konfrontieren würden. Gründe für ihre Zurückhaltung waren das Zögern, die Eltern zu konfrontieren, männliche Freundschaften nicht ruinieren zu wollen oder den Ausschluss durch die männliche Peergroup zu riskieren.

**Schlussfolgerung:** Lehrende müssen Wege finden, männlichen Universitätsstudenten dabei zu helfen, ihre Privilegien einzuordnen und sich ihrer Bereitschaft, sich auf unterdrückerisches Verhalten einzulassen, bewusst zu werden. Männer müssen auch verstehen, wie ihre Inaktionen systemische Unterdrückung aufrechterhalten und Umgebungen schaffen, in denen als Minderheit angesehene nicht gedeihen können. Empfehlungen für Lehrende der medizinischen Fakultät werden gegeben.

## 1. Introduction and literature

Ubiquitous media coverage of U.S. and European politicians suggests a veritable resurgence of vitriol, including racist, sexist, misogynistic, and homophobic language, attitudes, and behavior. In the U.S., the targets are people of color, immigrants, Muslims, as well as gay, lesbian, and transgender people; in Europe, the “other” are typically refugees, Jews, and people with migration background. 

Hateful behaviors are not exclusive actions of American or European politicians alone. They are, however, nearly exclusively male behaviors [10]. The racial and sexual parameters in which men operate are deeply imbedded in Western masculinities. American college campuses, not restricted to but also including medical colleges, are breeding grounds for these behaviors. A plethora of unacceptable bias-motivated incidents, including hate speech, assaults, and racist graffiti take place on U.S. college campuses with disturbing frequency. 

Most offenders of these actions are not only men; they are also white. American society has bestowed upon them the most social capital and privileges for which exist vast empirical evidence [19]. They do not question their whiteness or their masculinity as these identities are normative cultural traits against which members of minoritized communities are judged [4].

### 1.1. White men as campus climate creators

Campus climate can be defined as attitudes, behaviors, and practices that focus specifically on individual and group needs, abilities, and potential of all students, staff, and faculty [30]. Most campus climate research focuses on the racial or ethnic climate on college campuses [12], [33], [35], on the climate perceived by women [1], [25], and the climate perceived by community members with diverse genders and sexual orientations [5], [8], [30], [34]. 

Students, staff, and faculty from underserved groups report similar impressions of the climate at their institutions: on all predominantly white campuses, forms of racism exist inside and outside of the classroom. Unfriendly campus climates affect minoritized students negatively, including leading to stress, isolation, stereotyping, blunted cognitive or developmental outcomes, reduced engagement in educationally purposeful activities, or leaving college. In U.S. medical colleges, the dominant racial atmosphere is that of whiteness; painfully noticed by students of color and not interrogated by white students [36].

We strongly believe that white college men are the main creators of campus climates at predominantly white institutions. These climate creators include white male students, faculty, coaches, mentors, advisors, and supervisors. They also include administrators, the disproportionately white and male leadership of American universities, including presidents, their right-hand vice-presidents, provosts, and deans. Along with boards of trustees or regents, administrators represent the most power at the institution. Not only in the U.S., but also in Europe and Germany white men continue to be over-represented in the governing positions of colleges and universities. This also holds true for medical colleges. 

#### 1.2. White men and racist joking 

Among white college men joking is a ubiquitous behavior [6]. Unchallenged joking, objectification, and dehumanization may be harbingers for more serious forms of male violence [2]. Racist joking obfuscates the menacing fact that these performances are the vehicle to communicate ingrained bias for racial others [10], [28]. The joke, thus, is a representation of the systemic, centuries-old American white supremacy. Left unchecked, jokes are a sinister reminder of how American systemic and structural oppression continues to endure. 

Whites perform racial jokes in front of their friends or family to belong, to gain social capital, and to affect the attitudes and behaviors of other group members [28]. These performances occur in two spatial settings. In the frontstage (public spaces that are often multicultural) whites seem to get that joking, microaggressions, or overt racism are inappropriate. In the backstage (private, behind closed doors, and mostly white spaces), whites tell racial jokes in ways that are unchallenged and condoned. The racist representations in jokes are a cornerstone of the ways in which whites interact with racial others, including perennial tales of black men as oversexed, animalistic, or threatening humans [10]. 

To disrupt this pervasive oppression, straight white men specifically have to become vocal nonconformists to joking. All too frequently, however, challengers of inappropriate joking are criticized by the culprit or their peers: “Instead of the joke teller being held accountable, the challenger often must defend [their] intervention” ([28] p. 250). The common denominator in all of these acts are straight white men who either assert their oppressor role or their role of as passive bystander. 

#### 1.3. White men as bystanders

Straight white men on campus who create campus climates and who sanction or witness the inappropriate joking of peers must explore ways to hold each other accountable. This includes revealing the contexts in which white college men act as active or passive bystanders during incidents related to systemic oppression. 

Bystanders are individuals who observe actions that indicate oppression on campus, or in the community. Yet, bystander behaviors not only include active dissent of behavior; they can also include inaction, condoning, or supporting inappropriate behavior. 

The most important factor in predicting bystander behaviors in men may be their peer group. Despite their socialization as independent, stoic, and competitive, college men may actually prefer male friendships based in trust, emotional intimacy, and shared interest [29]. However, the norms-enforcing peer group may sway men to conceal their needs. 

As they compete for approval from peers, college men may consider performing masculinity in specific contexts that avoids expressing emotion, takes fewer risks in disclosing vulnerability, and desperately avoids appearing gay or feminine [18], [29], [32]. Performative masculinity includes joke-telling or name-calling, and boys grow up in settings where such behavior is widespread [26], [29]. 

In college, men may interact in a peer-enforced atmosphere and perceive other men are policing their behaviors. As a result, men analyze benefit and cost of confronting peers and being ousted by them [16], including the contexts of racist joking. This setting is potentially so norming that men remain inactive to gain (or at least not lose) social capital. Men who care about being part of a peer group and sense anxiety to conform will likely choose "laughing with the crowd" ([23], p. 514) over open confrontation and potential ostracism. 

The literature implies key inferences for educators, including those in the U.S. and Europe. Not only should we encourage men to investigate their needs for emotive male friendships, but we should also suggest they take risks in confronting friends for behaviors damaging to their own humanity as well as the humanity of minoritized individuals. 

#### 1.4. Theoretical framework

The Straight White College Men Project was grounded in the scholarship and theoretical underpinnings of critical whiteness. Critical whiteness studies interrogate and disrupt the invisible structures that create, develop, and perpetuate white supremacy and white privilege. Critical whiteness acknowledges that racism is inexorably linked to white supremacy and how white people may be complicit in racism unless they learn how to recognize their privileges and how to challenge the system that condones oppression. Whiteness studies call attention to how whites continue to benefit from racial privileges and how they have amassed economic and political power [21]. Whites perform whiteness by evading questions of power, by proclaiming color-blind attitudes, and by asserting that individual actions of good whites are enough to end systemic racism [14]. Considering oppression an individual choice is especially problematic in the critical whiteness paradigm: “When White people view only one level of whiteness, the individual level, they may feel powerless yet have greater relative power than racially minoritized people” ([3] p. 398). One of the aims of this paper is for readers, specifically straight white men, to understand that racism is not an individual human pathology, but a structural and systemic problem of a society. 

#### 1.5. Purpose 

This paper describes a portion of findings from the Straight White College Men Project, a large multi-institutional phenomenological focus group study with 92 straight white college male participants conducted across 10 different college campuses in the United States [35]. The purpose of the larger project was to explore and understand how participants perceived institutional and community diversity issues; and how they articulated their own perceived responsibility to engage in social change. 

For the purposes of this specific contribution that is limited in scope, we only report the findings from one focus group question assessing how participants responded or would respond to issues of racism on their campus or in their communities. The paper thus delivers lessons learned from engaging straight white college men in conversations on racism. 

## 2. Methods

### 2.1. Research sites

Ten four-year U.S. universities served as research sites for this project. All institutions were predominantly white and enrolled mostly female-identified students. Most U.S. regions are represented in the study; however, the majority of the sites were located in Midwest. 

#### 2.2. Sampling and data collection

We used purposeful criterion sampling strategies [27] and chose all research sites because of colleagues who could provide access to students. These expert nominators identified and, in some cases, signed students up for focus groups. Selection criteria included identifying as white, straight, and male, full-time undergraduate students. Data were collected between 2013 and 2016. 

We chose focus groups as the data collection method as they allow participants and moderators to co-create meaning of a specific experience [36]. A team of four researchers shared focus group moderation and each team member moderated focus groups alone (the author of this paper is the principal investigator of the study and the other researchers are no longer part of the team). Focus groups ranged in participants from 3 to 8 each, each group was digitally recorded and transcribed verbatim, and each lasted between 60 and 90 minutes. In sum, the researchers conducted 22 focus groups with straight white male college students at the 10 institutions. In total, 92 straight White men participated in the study, whose average age was 22. 

Focus group questions were derived from the literature on critical whiteness, masculinity, and social justice ally development. Sample questions in the focus groups included “How do you define diversity,” “What is your understanding of how oppression works,” “What is it like to be a straight white man on this campus,” and “What do you think is your responsibility to reduce issues of racism, sexism, or homophobia on your campus?” 

Removing researcher bias in qualitative research is nearly impossible, but the research team took the following steps to address these biases. First, moderators instructed participants on the nature and goals of the study, about wanting to hear differing perspectives, and about the participants’ freedom to answer or skip questions as they saw fit. This aimed to address potential groupthink or conformity to singular ideas [13]. Second, moderators did not correct potentially racist language of the participants as focus group moderators accepted all responses from participants during data collection and analysis [11]. Finally, during the focus groups, moderators did not take any notes to not potentially unnerve or alert the participants. Potential disagreements between moderators and participants may lead to participant withdrawal from further engagement [36]. 

To ensure trustworthiness, the research team performed member checks with participants [22] inviting them to review, authenticate, and critique a one-page document that included initial interpretations of the specific focus group data. We invited all participants to engage in the member checks and they either agreed with our interpretations or did not respond. 

#### 2.3. Data analysis and reporting

Krueger and Casey [20] argued analysis for focus groups should be systematic and sequential. Two research team members completed all of the data analysis, starting by open coding [9] aimed to discover expected and unexpected statements participants made about dissenting, challenging, or confronting racist behaviors in their environment. Axial coding [9] followed that involved classifying the data into larger categories. The larger study included more than 1,100 individual speech excerpts, 197 codes, 37 larger patterns or data units, and 13 overarching themes. For the purposes of this paper, I report two of themes that are specifically important in the context of bystanderism and racist performances:

1. Challenging family and friends; and,
2. fearing social consequences.

## 3. Results

### 3.1. Challenging family and friends

Participants wrestled with expressing if and how they would confront family members or friends on inappropriate language or behaviors. One of the participants said, "It's hard to tell your parents not to say something [other participants laughing]. They've been telling you what to do your entire life." 

The following conversation took place between one of the authors and a student who struggled confronting his parents on inappropriate language. Notice the difficulty with which he described the situation: 

Student: I come from a pretty conservative family and I've actually found myself catching my parents saying a little like off-topic humor. [I’m] like, “Get with the picture guys. It's the 21st century. You can't be saying that stuff.” 

Vianden: Is that tough to do?

Student: No, I mean it's not. They're not being super offensive so it's like, “Mom, don't say that.” It's like, “Make sure you don't say that in public” and then that's it. It's just a little banter between my parents and I, if anything.

Here, we sense the participant’s difficulty of being more direct with his parents as well as his attempts to downplay their comments as not offensive or their interactions as banter. 

An equally large set of data emerged from the focus groups on participant perceptions of challenging friends on racist language or behavior. During his focus group, Aaron shared this perception about confronting friends: 

What I’ve learned at high school is if you embarrass someone in front of their friends by calling them out, you have less of a chance of changing their heart than if you take them aside and explain to them. It’s something more loving. Challenging someone openly in front of others, people tend to get defensive.

Aaron’s description of weighing his dissent seems to imply fault in the confronter, not the originator of the behavior. 

Max expressed a bit of resignation in describing the decision-making process he considers when possibly challenging joking, language, or behavior of family:

If you’re the only one that’s going to stand up, like at home, I know for a fact I’ll be the only one to ever say anything, cause my whole town is racist and doesn’t care what anyone else thinks.

If Max's town is the way he describes, it might be a perfect place to begin sowing seeds of challenging racist behavior or language. Yet, we also understand why he may be reticent to do so on his own. One key would be for him to identify other white male family, neighbors, or friends, who think similarly about their community. Those individuals exist, but the task of identifying them may seem too arduous for Max. 

#### 3.2. Fearing social consequences

Next, we turn to why it may be difficult for men to challenge family and friends who use inappropriate language. Consider Leo's comment: 

It’s hard to speak up. Especially if you don’t like conflict. If you don’t want to be labeled as like, [for] lack of a better term, the dick. Like you don’t want everybody to think, “Well, that guy doesn’t have a sense of humor.” Then you get labeled yourself, and nobody really wants to deal with you.

Just how important the peer group is to college men is evident in this statement by Jamie:

For me personally, it’s easier to confront a stranger than a friend, just because I don’t really care about their opinion. They mean nothing to me. Yeah, they mean nothing to me, whereas my friends [and] their opinion of me, just mean about everything.

Discussing why men may struggle to confront inappropriate language by friends, James shared this comment:

If you’re in your friend group and you’re the only guy who [throws] the first wet blanket on the joke that everybody else gets. There could be serious repercussions [if you're] the guy who ruins jokes every time. You don’t want to be that guy.

Brad made this statement about being liked by friends: “We should be able to [confront], but we won’t. We want to go along with it, we want them to like us in a sense, and by laughing they think that [we like them].” For Abe confronting friends would mean to jeopardize the relationship: “It’s easier to [stand up to] a stranger than to say it to someone you have more of a connection with. [The] relationship with [your friend] is now on the line because you’re criticizing what they said.” For Zane, confronting friends on language could potentially end the friendship: 

I think it’s a hard thing to confront friends [when] they make a comment that crosses the line. Because you don’t want to say something and then they get really angry at you. You don’t want to make things weird or awkward, or potentially ruin a friendship because some people are just that sensitive.

## 4. Discussion and recommendations for medical education

Many of the findings reported here are supported by the literature on men and masculinities, men’s bystander behaviors, and the reticence of men to confront members of their peer group on inappropriate language or behaviors. Men’s trepidations about showing solidarity to oppressed people and confronting inappropriate language or joking are common. Human beings who are part of a peer group from which they seek support, affirmation, and validation will likely not risk that friendship by actions not supported by the group [16], [24]. Many of our decisions are driven by how we perceive our reference group will react to what we do, rather than how the group actually reacts. Men specifically may be anxious that their outward dissension will get them ousted from their reference group [18]. So, we remain silent even when witnessing what we know is inappropriate peer behavior [17]. As we saw in the findings and as the literature indicates, the challenger of the joke has often must defend himself, while the perpetrators go unchecked [28]. The fear that “the violence just might be turned against them if they voice their opposition too vehemently” ([17], p. 61) is one reason why men may not challenge their peers as much as we might expect. Educators have a lot of work to do with college men who assume someone would end a friendship because they have been confronted on inappropriate language. 

We often fail to understand why assumed assertive, competitive, or self-sufficient men seem to have trouble challenging others’ inappropriate behaviors. We frequently miss the complex and intricate processes at work in the male peer group, and we tend to underestimate the emotional connection men have with one another because some assume men are bereft of emotion [32].

You may say, “what does this have to do with medical education in a German or European context?” In Europe and Germany, medicine has been dominated by white men and in which white men have power, make the rules, and assess the education and development of all other members of the medical community. In her famous essay, “Cloning the Physician,” Philomena Essed [8] criticized the medical field for its apparent desire to reproduce the white European male physician who rises to a position of influence, and who continues to homogenize, preserve, and promote a culture of other powerful white male physicians. Cloning happens by dominating the medical field with white male norms and then attributing more medical competence to such norms and men over non-white men or women [8].

If the findings of the present research study relate to a German or European medical school context, white male German medical students will struggle similarly to challenge or dissent inappropriate language from peers, professors, or university staff. This inaction, in turn, has the potential to perpetuate a white patriarchal system in which women, people of color, and members of other less-dominant social groups are minoritized. In classrooms, labs, hospitals, or doctor’s offices where students of medicine train, a welcoming climate needs to exist for minoritized individuals to thrive. Several accounts exist of how such places are typically racist, at least in the U.S. [26]. Creating a welcoming climate is not automatic. It takes work by the most socially dominant and powerful members of the medical community. 

Consider our following recommendations for German and European educators who work with white men in schools of medicine.

### 4.1. Develop responsibility for social action

Most participants of the current study could not articulate an immediate commitment to challenge language or joking of family or friends. These men, based on their social capital and privileges in society will one day hold leadership positions in which they could affect positive change. The same is true for students in Europe and Germany who are seeking positions in medicine. Given this context, it is imperative that educators challenge white male students to interrogate their privileges and understand the roles they play in a diverse world. The problem is that medical colleges, which are typically apart from the undergraduate or graduate colleges of the university, are run by faculty and physicians who may be part of a homogenous white male culture and who have not interrogated their own privilege or oppressive behaviors. It may be a stretch, but each medical college must identify educators who train medical instructors, physicians, and students and who hold them accountable to educating and behaving in non-oppressive ways. White male educators and students in schools of medicine must recognize how their privileges have the potential of keeping peers with minoritized identities without opportunities to advance. 

#### 4.2. Recognize personal sphere of influence 

It is not surprising that our participants may have struggled challenging potentially inappropriate language and behavior stemming from family and friends. The people we challenge when we say, “please stop” are those with whom we tend to have the most special relationships, and a stronger bond than with anyone else. Their affirmation and love matter deeply and many of us have been taught to respect our elders. Yet, it is important to find ways to exert influence in one's personal sphere if white men are to develop solidarity with others and to advocate for social justice. Because of our strong relationships in the personal sphere, challenging here may be most effective. Educators should involve white men in discussions, exercises, and activities that enable them to build skills to affect change among those to whom they are closest. 

#### 4.3. Challenge peer norms 

We appreciate the vulnerability of men to fully disclose their reticence to challenge inappropriate behavior of their peers. This has non-trivial implications for learning and development of college students, as well as for teaching, advising, supervising, and mentoring. Men, who are socialized to be rough and tough on one hand, are also forthright about their inability about leading the charge to disrupt racist language and behaviors. In medical schools that may socialize students-becoming-physicians to refrain from disclosing personal or emotional information, not addressing dissent of inappropriate behaviors may exacerbate privilege and oppression and further burden those “othered” by the profession and institution. White college men are not incapable of building solidarity with others or unfit to advocate against oppression; yet, it will require more specific reaching and teaching by instructors and advisors. Educators must engage white men in conversations about the power of the peer group and together construct knowledge and build skills around appropriate confrontation of toxic masculinity. In a discussion moderated by a skilled educator, men can learn how to challenge other men, and those challenged can learn to listen and understand that their behaviors are hurting others as well as themselves. 

## 5. Conclusion

This article described white college men’s difficulties to confront racist joking of family members and friends. A reason for refraining from confrontation included fearing social consequences from their family or peer group. 

Against this backdrop I attempted to provide “lessons learned” from engaging with straight white college men on the topic of racism, and to issue a call for action for white college men and their educators. Why should white men in college or university become more active and stand up to, compared to passively witnessing discrimination and oppression in the United States, Europe, or Germany? Because we're in a social position of power, because our humanity requires it, and because the outcome is a more socially-just society, one from which all citizens benefit. If school of medicine educators are successful in engaging white men in this work, White men will improve their profession, their immediate spheres of influence and communities, as well as the society of which they are part. 

## Competing interests

The authors declare that they have no competing interests. 
